# Advanced Silicon-on-Insulator: Crystalline Silicon on Atomic Layer Deposited Beryllium Oxide

**DOI:** 10.1038/s41598-017-13693-6

**Published:** 2017-10-16

**Authors:** Seung Min Lee, Jung Hwan Yum, Eric S. Larsen, Woo Chul Lee, Seong Keun Kim, Christopher W. Bielawski, Jungwoo Oh

**Affiliations:** 1 School of Integrated Technology, Yonsei University, Incheon, 21983 Republic of Korea; 2 Yonsei Institute of Convergence Technology, Incheon, 21983 Republic of Korea; 3 Center for Multidimensional Carbon Materials (CMCM), Institute for Basic Science (IBS), Ulsan, 44919 Republic of Korea; 4 Department of Chemistry, Ulsan National Institute of Science and Technology (UNIST), Ulsan, 44919 Republic of Korea; 5 Center for Electronic Materials, Korea Institute of Science and Technology (KIST), Seoul, 20792 Republic of Korea; 6 Department of Energy Engineering, UNIST, Ulsan, 44919 Republic of Korea

## Abstract

Silicon-on-insulator (SOI) technology improves the performance of devices by reducing parasitic capacitance. Devices based on SOI or silicon-on-sapphire technology are primarily used in high-performance radio frequency (RF) and radiation sensitive applications as well as for reducing the short channel effects in microelectronic devices. Despite their advantages, the high substrate cost and overheating problems associated with complexities in substrate fabrication as well as the low thermal conductivity of silicon oxide prevent broad applications of this technology. To overcome these challenges, we describe a new approach of using beryllium oxide (BeO). The use of atomic layer deposition (ALD) for producing this material results in lowering the SOI wafer production cost. Furthermore, the use of BeO exhibiting a high thermal conductivity might minimize the self-heating issues. We show that crystalline Si can be grown on ALD BeO and the resultant devices exhibit potential for use in advanced SOI technology applications.

## Introduction

Silicon on sapphire (SOS), consisting of a thin layer of Si grown on a sapphire (Al_2_O_3_) wafer, is a material that employs the silicon-on-insulator (SOI) complementary metal–oxide–semiconductor (CMOS) technology commonly used for the fabrication of integrated circuits. The importance of SOS stems from its insulating characteristics, which help to eliminate the parasitic drain capacitance, enhance transistor performance by reducing capacitor charge and discharge cycles, and protect critical circuit elements^1,2^. Furthermore, the drain capacitor of bulk Si operates in a non-linear manner that facilitates the rapid depletion of capacitance between two semiconductor layers^3,4^. SOS does not exhibit such a non-linear behavior, thereby exhibiting a higher high-frequency performance than bulk Si. Wireless radio frequency (RF) applications are a prime example of a contemporary technology that benefits from SOS^5–7^. Despite the significant advantages of SOS, the high cost of sapphire substrates and the presence of defects have challenged their commercial development^8,9^. To solve these problems, various methods including the deposition of Si on crystalline oxides, such as cubic spinel, have been explored^10,11^. Unfortunately, the lattice mismatch of crystalline oxides^12^ and Si is typically large, leading to the formation of defects during the heteroepitaxial SOI process.

**Figure 1:**
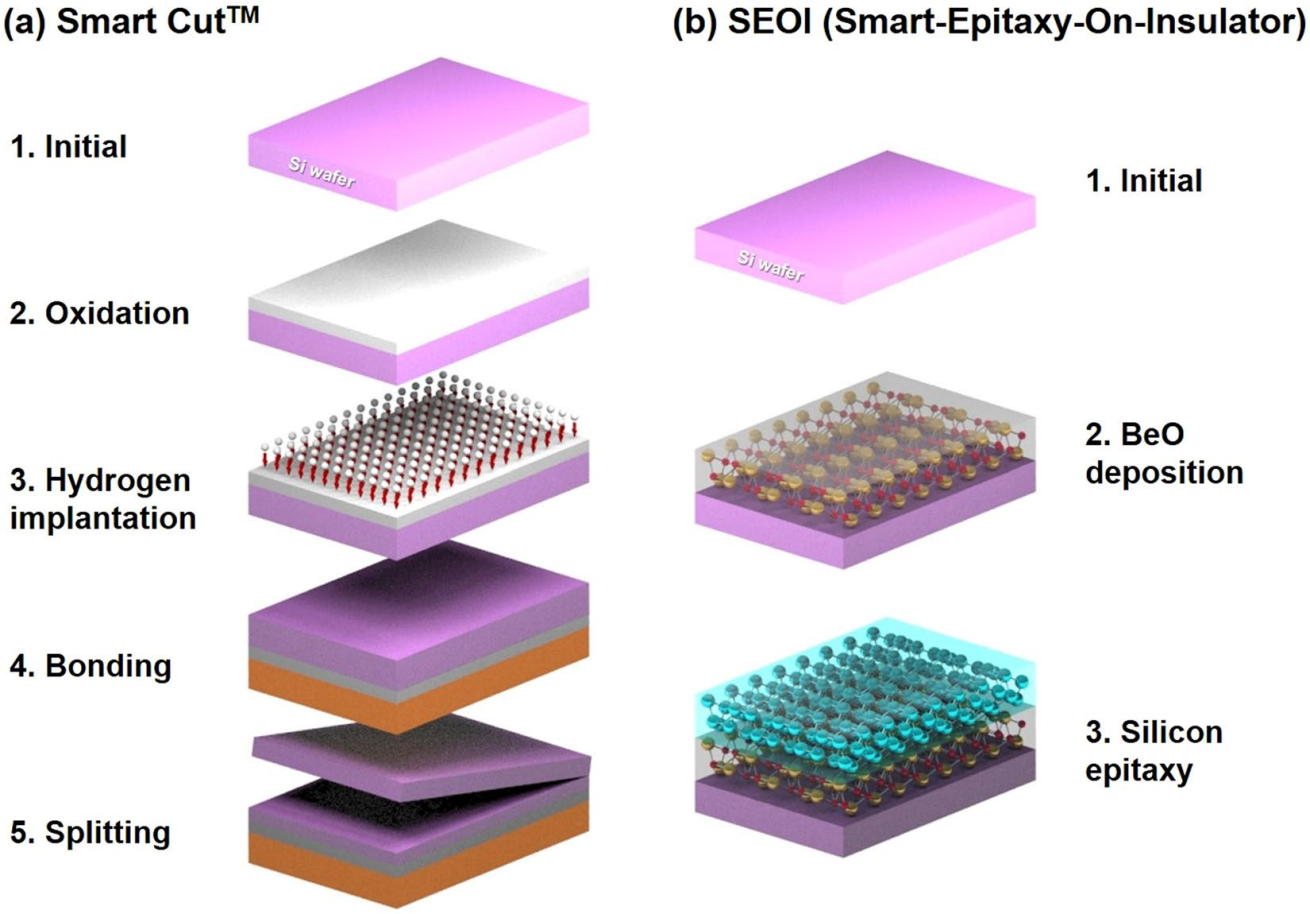
Schematic diagram of (**a**) smart-cut wafer bonding method and (**b**) smart epi-Si growth on ALD BeO. The smart-cut process is comprised of four steps: 1) Oxidation to form a buried oxide on wafer A (purple); 2) Hydrogen implantation into wafer A to create a weakened layer; 3) Bonding and annealing to form the chemical bonds between wafer A and handle wafer B (brown); 4) Splitting to cleave the weakened layer from wafer A. The SEOI process is comprised of two steps: 1) Deposition of crystalline BeO to form a buried oxide; 2) Epitaxial growth of silicon to create a thin film layer.

Wafer bonding is often used as an alternative to the aforementioned strategy. Figure 1a illustrates the “smart-cut wafer” bonding method^13^. An insulating layer is formed by directly bonding the oxidized Si to a second substrate^14,15^. After removing the second substrate using ion implantation and controlled stripping, Si is formed on the top layer in a controlled manner. While this method is effective, the process is complex and the cost of the resultant SOI wafers is typically 6 to 8 times higher than that of the conventional Si wafers. In addition, the presence of the silicon oxide insulating layer causes self-heating problems by preventing the transfer of heat generated during ultra-fast CMOS device operations^16,17^.

To minimize the deleterious effects caused by self-heating, oxide materials with a higher thermal conductivity than that of sapphire (~23–70 W/K.m) could be used^18^. BeO exhibits a high thermal conductivity (330 W/K.m), and highly crystalline BeO thin films can be grown on Si (100) substrates^19–22^. Moreover, BeO exhibits excellent properties suitable for SOI technology, including high electrical resistivity, radiation resistance, low phonon scattering, and low structural defect density. In addition, atomic layer deposition (ALD) can be employed for producing high quality BeO films with well-controlled thickness in a cost-effective manner^23,24^. The advantageous of ALD include the stoichiometric control, excellent reproducibility, and low defect density^25–27^. In order to overcome the disadvantages of the smart-cut wafer bonding method in SOI technology, we combine the advantages of ALD and the excellent physical properties of BeO for the first time as illustrated in Fig. 1b.

## Results and Discussion

**Figure 2:**
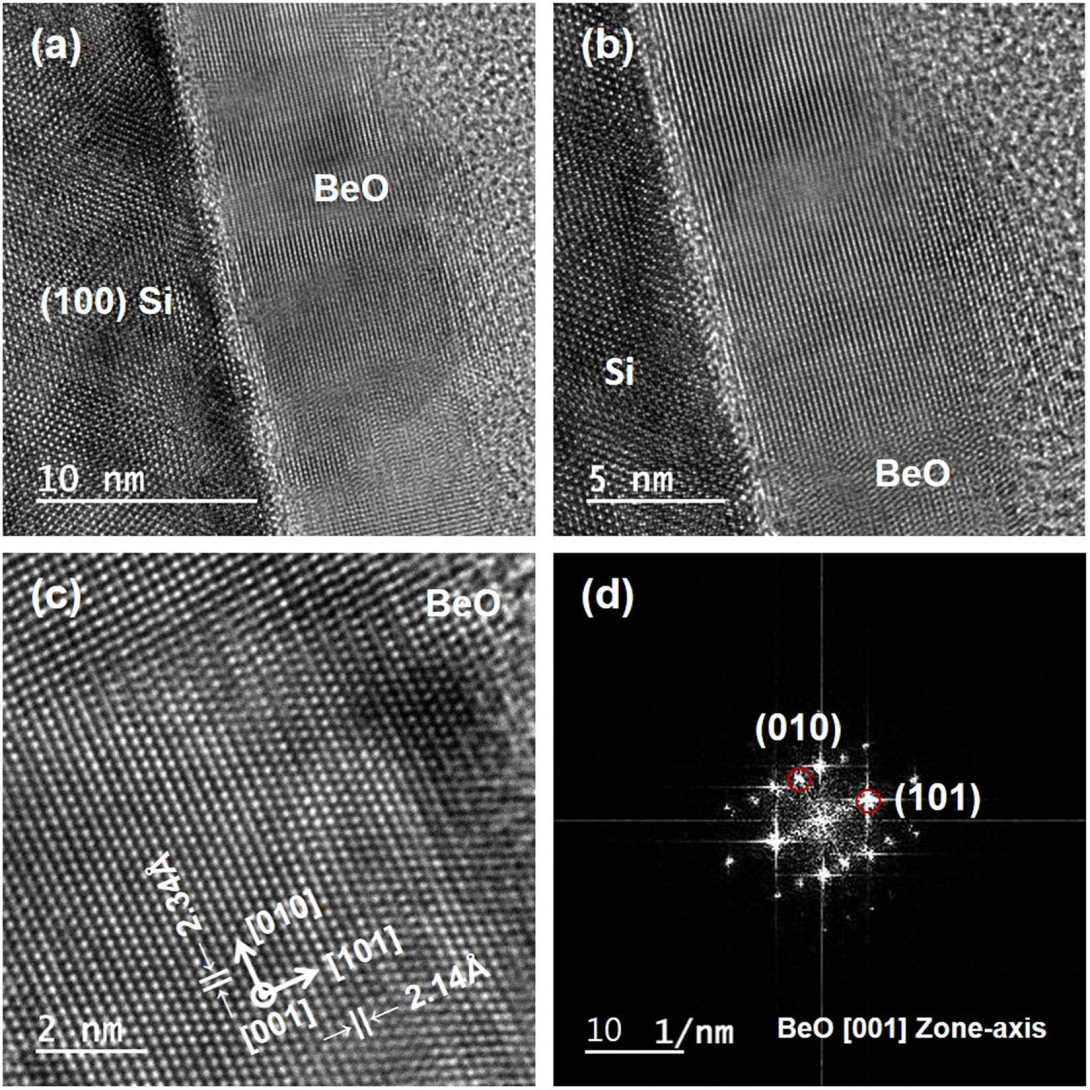
Cross-sectional high-resolution TEM of ALD-grown BeO film on Si (100) at (**a**) 10, (**b**) 5, and (**c**) 2 nm-scale magnifications. (**d**) TEM FFT diffraction pattern of ALD BeO. High-quality crystalline BeO (101) was grown on Si (100). The inter-planar spacing (d-spacing) results in (**c**) and FFT diffraction patterns confirm that the growth directions of the BeO film are [010] in-plane and [101] out-of-plane.

After cleaning a Si substrate surface with an aqueous solution of HF (1%) for 1 min, BeO was deposited on Si (100) at 250 °C employing ALD using dimethylberyllium as the precursor (see Supplementary Figures S1 and S2). Figure 2a shows a cross-sectional TEM image of the crystalline ALD BeO thin film grown on a Si (100) substrate, clearly showing three layers corresponding to the Si substrate, the BeO layer, and the epoxy adhesive. BeO is crystalline over a considerably wide range. The thickness of the crystalline BeO film is ~12 nm, which is in good agreement with the results obtained from the multiple wavelength ellipsometry measurements. To further investigate the crystallinity of BeO, cross-sectional TEM images were recorded at different magnifications (see Fig. 2b and c). The cross-sectional TEM images show that the BeO film is highly crystalline and the crystal size deduced from the TEM data is in the range of 20–30 nm. The direction of the incident electron beam is parallel to the underlying [110] and [001] directions of Si and BeO, respectively. The BeO film formed on the Si substrate exhibits a d-spacing of 2.14 Å (see Fig. 2c) as measured from the lattice image. The standard d-spacing value of the (101) plane of BeO is 2.06 Å according to the joint committee on the powder diffraction standard (JCPDS) database. Thus, we conclude that (101) is most likely the growth direction for the ALD BeO film growth on the Si substrate. A TEM finite Fourier transform (FFT) diffraction pattern attributable to (001) wurtzite BeO is shown in Fig. 2d.

Epitaxial Si layers were deposited in a commercially available, cold-wall stainless steel UHV/CVD reactor with a substrate temperature of 700 °C. The reactor back-pressure was less than 5 × 10^−9^ Torr and the pressure was maintained to be less than 7 × 10^−5^ Torr during the deposition process to maximize the precursor mean free path and minimize the chemical reactions in the vapor phase. Undiluted, high-purity Si_2_H_6_ was injected at 10 standard cubic centimeters per minute (sccm) without purifiers through calibrated mass flow controllers and high-speed turbo-molecular pumps to ensure the pressure to be less than 7 × 10^−5^ Torr. To minimize the amounts of contaminants introduced during wafer loading, the deposition chamber was attached to a UHV transfer chamber platform equipped with UHV load locks and compatible wafer handling robots.

**Figure 3:**
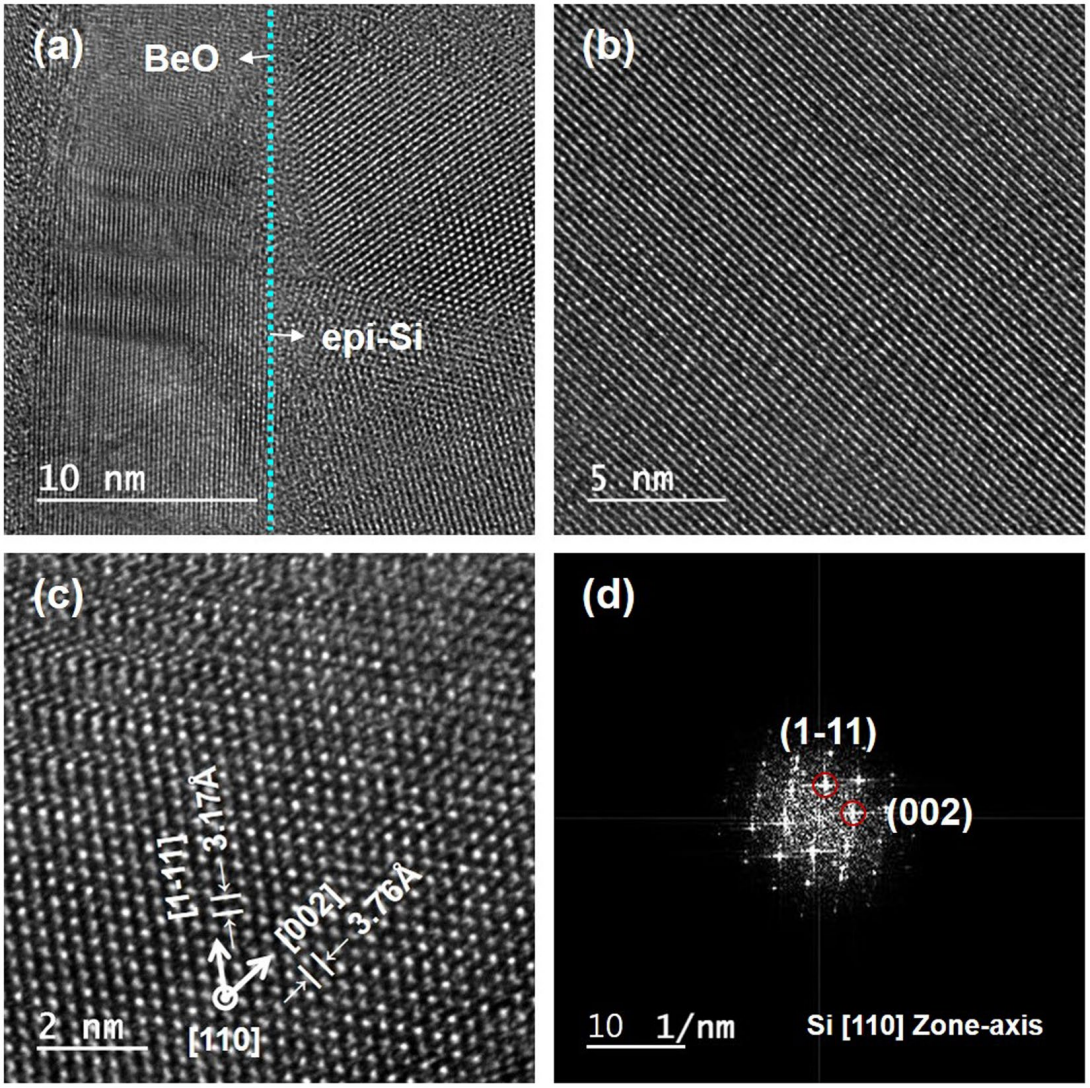
Cross-sectional high-resolution TEM image of UHV/CVD epi-Si on ALD-grown BeO at (**a**) 10 (**b**) 5, and (**c**) 2 nm-scale magnifications. (**d**) TEM FFT diffraction pattern of epi-Si. A polycrystalline Si layer was epitaxially grown with tilt on a BeO thin film. The d-spacing results in (**c**) and FFT diffraction patterns identify that the growth direction of epitaxial silicon is [1–11] out-of-plane.

Figure 3a shows a cross-sectional TEM image of the epitaxially grown Si film (epi Si) on an ALD BeO film, where layers of the Si substrate, BeO film, epi Si film, and epoxy adhesive are clearly observed. To investigate the crystallinity of the epi Si layer, cross-sectional TEM images were recorded at different magnifications (Fig. 3b,c). The cross-sectional TEM image (Fig. 2b,c) shows that the epi Si layer grown on the ALD-grown BeO thin film is highly crystalline, and crystal dimensions of at least 20–30 nm are obtained using TEM. Moreover, the diffraction pattern corresponding to the (110) epi Si layer is clearly observed (Fig. 3d). The direction of the incident electron beam is parallel to the [001] and [110] directions of BeO and epi Si, respectively. The d-spacing of the Si epi grown on the BeO thin film is measured to be 3.17 Å from the lattice image, which is close to that of the Si (111) plane according to JCPDS database (3.14 Å). Thus, Si (111) is most likely the growth direction for UHV/CVD epi Si grown on the ALD BeO thin film. However, because the d-spacing of the epi Si (111) is slightly higher than that of the single crystalline Si (111) substrate, tensile stress could be applied to epi Si on ALD BeO.

**Figure 4:**
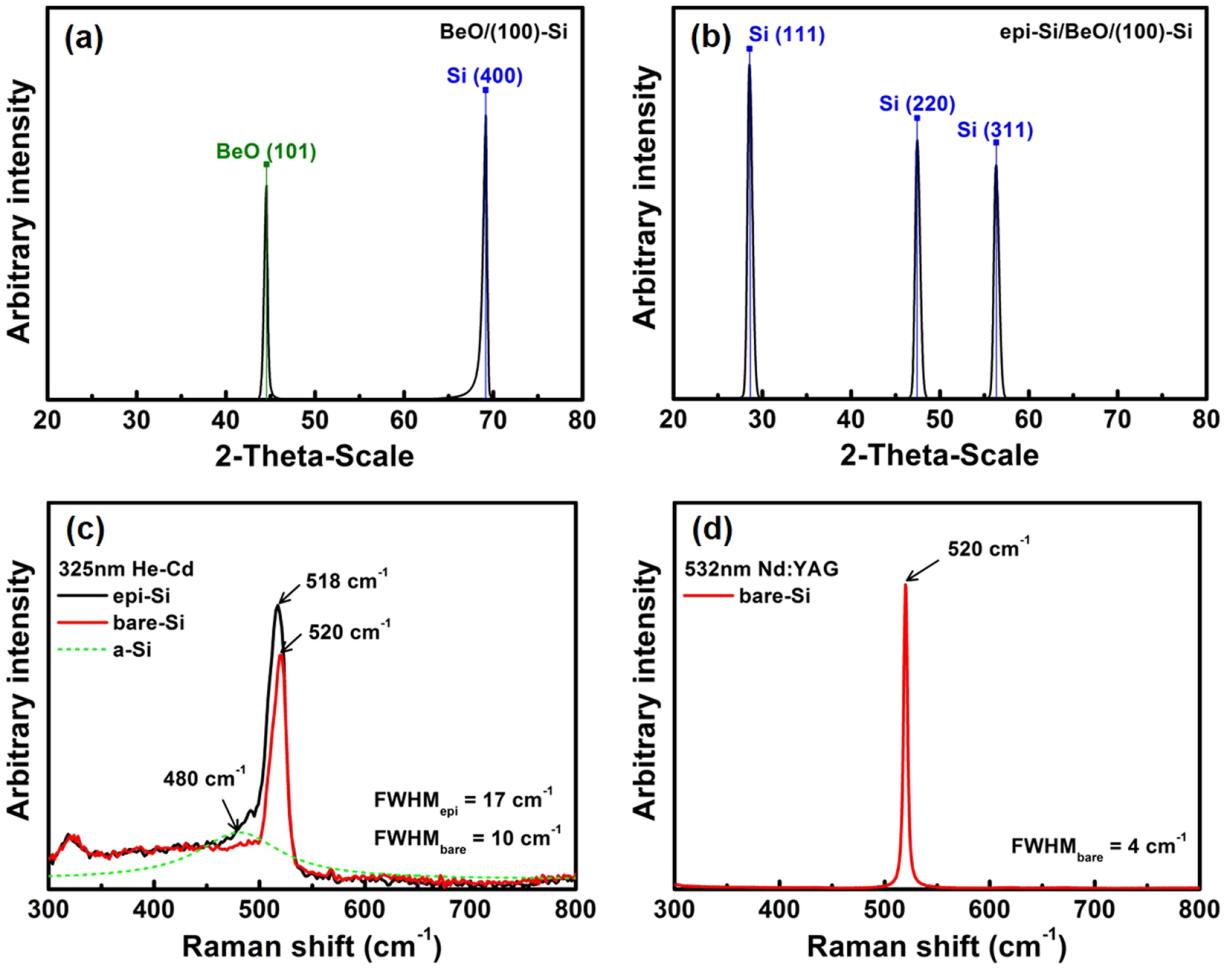
(**a**) XRD 2θ/ω scans of ALD BeO; the crystal orientation of BeO is only (101) phase. (**b**) XRD 2θ/ω scans of UHV/CVD epi-Si; the polycrystalline Si mainly has a (111) crystal orientation and a relatively small (110) phase. (**c**) Raman spectrum of epi-Si and bare-Si with 325-nm He-Cd laser; the epi-Si film showed a sharp peak at 518 cm^−1^ and a small amorphous-Si peak at 480 cm^−1^. (**d**) Raman spectrum of bare-Si with 532 nm Nd:YAG laser; the FWHM of bare-Si was about 4 cm^−1^, which is close to the literature (3 cm^−1^).

High-resolution X-ray diffractometry may be used to accurately assess the crystal orientation and lattice spacing. Therefore, high angle 2θ/ω X-ray diffraction measurements were performed on the epi Si (50 nm) grown on ALD BeO (12 nm). Figure 4 (a) shows the XRD spectrum of the ALD BeO film grown on the (100) Si substrate, and Fig. 4 (b) shows the XRD spectrum of epi Si grown on the ALD BeO thin film. The XRD data indicate that the ALD BeO film grows on the Si substrate mainly with the (101) crystal orientation, which is in good agreement with the TEM and FFT results (see Fig. 2). Subsequently, epi Si was grown primarily in the (111) crystal direction on the ALD BeO film. The crystal size of the (110) plane is relatively small. The crystallinity of the epi Si thin film was also investigated using Raman spectroscopy with a 325-nm He-Cd laser. The 325-nm laser was preferred to scan the 50-nm thickness of the epi Si top layer due to its shorter penetration depth. As shown in Fig. 4c, the epi Si showed a sharp peak at 518 cm-1, which is close to the bare single crystal Si (100) peak at 520 cm-1. The amorphous Si peak at ~480 cm-1 was not significant after the deconvolution of the epi Si peak. These results collectively suggested to us that the crystallinity of the epi Si film deposited on the ALD BeO thin film was very high. The Raman signal positioned on the epi Si film was shifted by 2 cm-1, and thus, it can be converted to strain using the following equation^28^.

1 $${\rm{\Delta }}{\rm{\omega }}=-1.6{\rm{\tau }}\,(c{m}^{-1}GP{a}^{-1})$$

where $\triangle {\rm{\omega }}$ is the change in the Raman peak position, and the positive value ${\rm{\tau }}$ corresponds to tensile stress. Thus, the peak position reflects the tensile stress on the epi Si film, which might arise due to lattice mismatch or thermal expansion coefficient difference between the epi Si and the ALD BeO film. The full width at half maximum (FWHM) of the epi Si and bare Si Raman peaks were measured to be 17 cm^−1^ and 10 cm^−1^, respectively. The more broadening of epi Si peak may be due to the small portion of amorphous Si peak. Generally, the FWHM of crystal Si Raman peak is known to be about 3 cm^−1^. However, the FWHM of bare Si Raman peak measured by 325 nm laser has wide value because the intensity of Raman peak is low due to the reduction of excited optical phonons by the shallow penetration depth. To confirm the FWHM of bare Si, an additional Raman analysis was performed to bare Si using a 532 nm Nd:YAG laser (having 760 nm penetration depth), showing that the FWHM was 4 cm^−1^ (see Fig. 4d).

**Figure 5:**
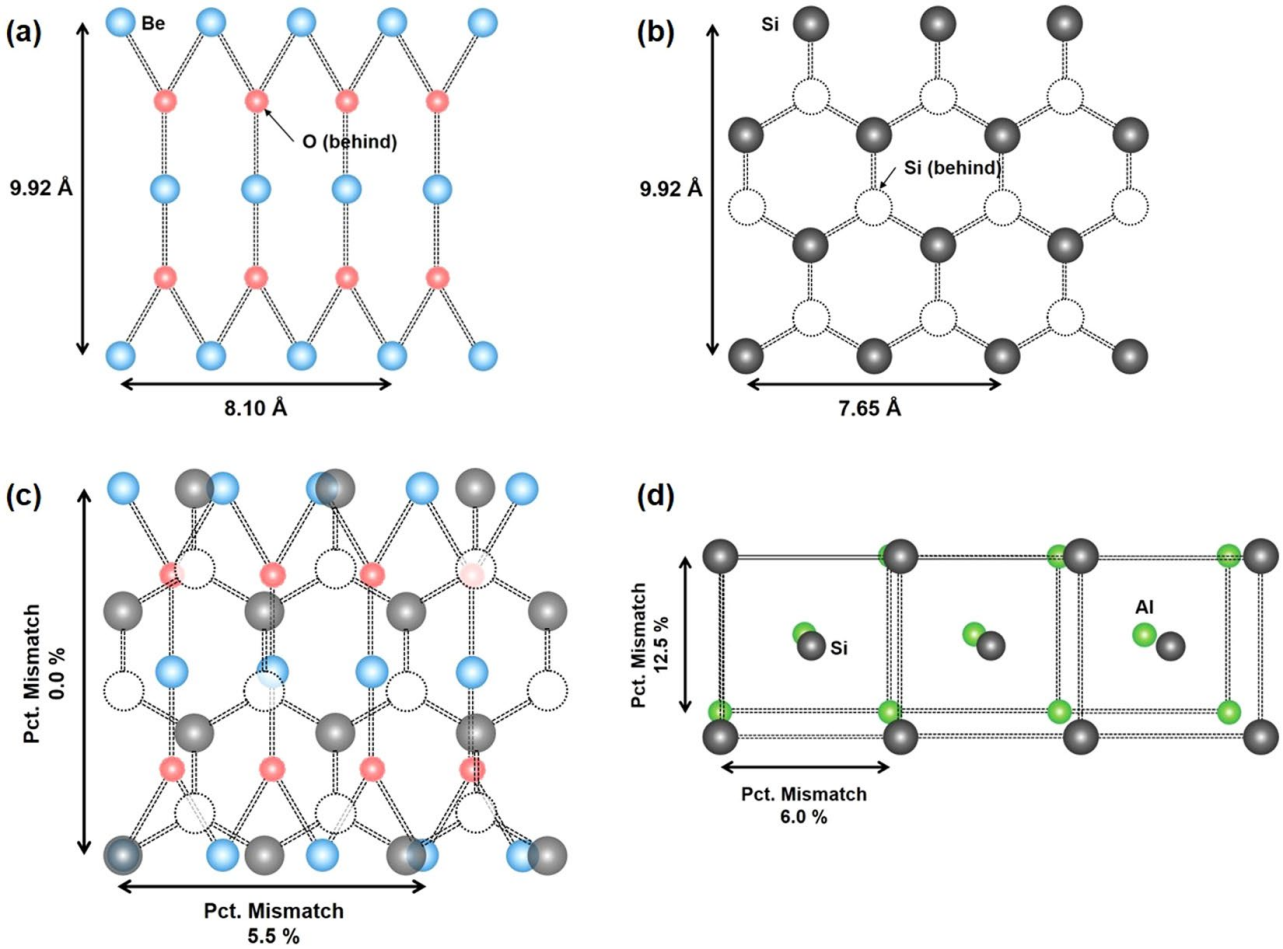
Atomic configuration of (**a**) BeO (101), (**b**) Si (111), and (**c**) two-dimensional overlay of Si on ALD BeO. The 3(Be):2(Si) domain-matching ratio has a mismatch of 5.5% in the horizontal direction, thus inducing uniaxial tensile strain in the BeO film. (**d**) Atomic configuration of Si on sapphire; the lattice mismatch between Si and Al is 6.0% in the horizontal direction and 12.5% in the vertical direction.

It has been reported that epitaxy growth occurs when there is a match between the atoms of the deposited film and the metal ion sites of the seed plane, and this matching should not be limited to a one-to-one correspondence between the sites in the two lattices^11^ (See Supplementary Figure S3). Once a crystalline relationship between the film and the seed layer is established, it is possible to determine the suitability of an atomic model for the ALD BeO and the epi Si heterojunction system. Atomic configurations of BeO (101) and epi Si (111) are depicted in Fig. 5a,b, respectively, based on the obtained TEM, FFT, and XRD results. BeO (101) exhibits a layered structure (Fig. 5a) and its surface is known to be terminated with Be or O atoms, in consistent with the data presented in Fig. 2c. To further validate our model, a Si (111) plane was superimposed on the (101) lattice of Be by preparing 2D overlays of the respective atom positions. If the ratio of the spacing in Si to that in Be is considered as 2:3 rather than 1:1, a lattice site mismatch of within 5.5% is obtained in the horizontal direction. In this situation, the Be lattice may induce tensile stress on the Si lattice, in consistent with the Raman results. In the vertical direction, the spacing values appear to be well matched with a ratio of Si to Be of 3:2. These results are superior to those obtained using SOS as shown in Fig. 5d (6% mismatch in horizontal direction and 12.5% mismatch in vertical direction). While the assumption of 1:1 lattice match is common, it has been reported that it not a necessary requirement for the epitaxy growth to occur^10,11^.

## Conclusion

In summary, we show that highly crystalline films of Si may be epitaxially grown from thin films of ALD grown BeO to afford layers with good crystallographic relationships and a low mismatch. The use of the simple ALD process to grow the aforementioned films of BeO causes the lowering of the cost associated with the production of SOI wafers, and the use of BeO with a high thermal conductivity may solve the intrinsic self-heating problems of silicon oxide. Therefore, films of epitaxial Si grown on ALD BeO can be employed for solving the current challenges in the contemporary SOI technology, and they could be used for advanced radio frequency and radiation sensitive applications as well as for reducing the short channel effects observed in microelectronic devices.

## Methods

### ALD parameters

BeO was deposited using an Atomic Classic ALD, Loadlock Module, CN1 with a reaction chamber temperature of 250 °C, precursor sublimation temperature of 110 °C, Be pulse of 3 s, H_2_O pulse of 1 s, Be purge of 20 s, H_2_O purge of 30 s, and chamber pressure of 0.8 Torr by performing 150 cycles at 0.8 Å/cycle.

### TEM measurements

TEM images were obtained using a JEOL JEM-ARM 200 F system. A Schottky field-emission electron beam was generated at 80–200 kV (magnification: 50 to 2,000,000 × , resolution: 0.1 nm). A focused ion beam (JIB-4601F) was used on electronically transparent samples that were obtained using a Disco 321 DAT dicing saw.

### XRD measurements

X-ray diffraction analyses were carried out using a Rigaku SmartLab system. The X-ray tube was operated at 20–60 kV and 60 mA with CuK_α_ X-rays. A scan axis of 2-theta was employed, and 2-theta values in the range of 20°–80° were used with a step width of 0.02° and scan speed/duration of 1.00°/min.

### Raman spectroscopy

All spectra were collected using a Horiba-LabRAM ARAMIS Raman microscope equipped with a 325 nm He-Cd laser, full range grating, and motorized stage. The instrument was operated using the Thermo Scientific OMNIC 8 software suite. OMNIC™ Atlμs™ mapping software was used to collect and analyze the data.

## Electronic supplementary material

Supplementary Information
