# Supplementary material for: Advanced Silicon-on-Insulator: Crystalline Silicon on Atomic Layer Deposited Beryllium Oxide
Source: Sci Rep. 2017 Oct 16;7:13205. doi: 10.1038/s41598-017-13693-6 (PMC5643296; doi:10.1038/s41598-017-13693-6)
Supplement: Supplementary file 1 — Supplementary Information [file 41598_2017_13693_MOESM1_ESM.doc]

**Supplementary Information**

**Advanced Silicon-on-Insulators: Crystalline Silicon on Atomic Layer Deposited Beryllium Oxide**

Seung Min Lee^1,2,+^, Jung Hwan Yum^3,4,+^, Eric S. Larsen^3,4^, Woo Chul Lee^5^, Seong Keun Kim^5^, Christopher W. Bielawski^3,4,6*^ and Jungwoo Oh^1,2*^

^1^School of Integrated Technology, Yonsei University, Incheon, 21983, Republic of Korea

^2^Yonsei Institute of Convergence Technology, Incheon, 21983, Republic of Korea

^3^Center for Multidimensional Carbon Materials (CMCM), Institute for Basic Science (IBS), Ulsan, 44919, Republic of Korea

^4^Department of Chemistry, Ulsan National Institute of Science and Technology (UNIST), Ulsan, 44919, Republic of Korea

^5^Center for Electronic Materials, Korea Institute of Science and Technology (KIST), Seoul, 20792, Republic of Korea

^6^Department of Energy Engineering, UNIST, Ulsan, 44919, Republic of Korea Republic of Korea.

Correspondence: Christopher W Bielawski (bielawskilab@gmail.com) and Jungwoo Oh (jungwoo.oh@yonsei.ac.kr)

**1. Synthesis process of ALD precursor**


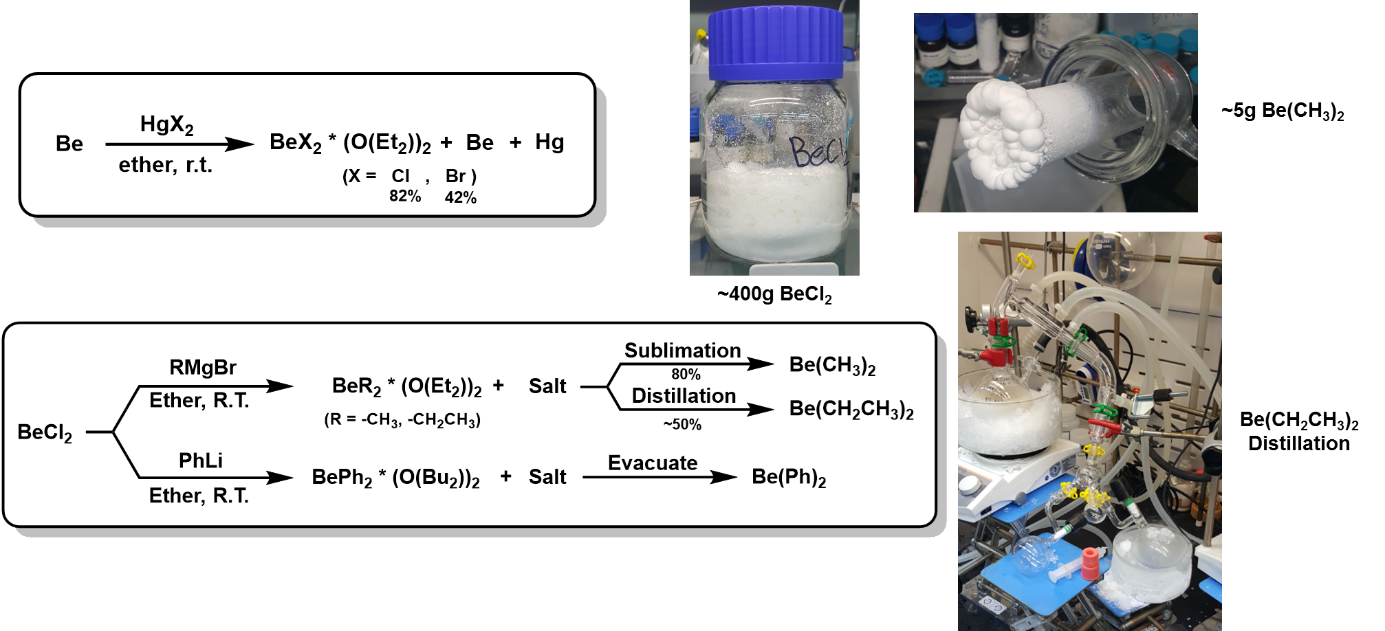


Figure S1. Dimethylberyllium and diethylberyllium precursor synthesis process.

**General Considerations.** The reactions were performed in a Vacuum Atmospheres Company drybox or using Schlenk techniques under an inert atmosphere of N_2_. All solvents were degassed and dried using a Vacuum Atmospheres Company solvent purification system prior to use. Beryllium metal and mercuric chloride were purchased from commercial suppliers and used without further purification. ^1^H and ^13^C NMR spectra were recorded using a Bruker 400 MHz or a Varian 600 MHz spectrometer. Chemical shift (δ) values are given in ppm and are referenced to TMS using the residual solvent (^1^H: C_6_D_6_, 7.16 ppm; C_5_D_5_N, 8.74 ppm; ^13^C: C_6_D_6_, 128.06 ppm; C_5_D_5_N, 150.35 ppm) or acquired in diethyl ether (Et_2_O) with an internal standard of C_6_D_6_ and externally referenced to BeSO_4_ in D_2_O (^9^Be: 0.00 ppm). Warning: beryllium and its related compounds are toxic and should be handled with care.

**First step: BeCl_2_(Et_2_O)_2_ synthesis:** After slowly adding HgCl_2_ (50 g, 0.184 mol) to a dispersion of Be powder (2.76 g, 0.306 mol) in Et_2_O (1 L) over a period of 1 h, the resulting mixture was stirred overnight and then filtered to remove residual mercury and unreacted Be. The collected filtrate was placed under dynamic vacuum to afford the desired compound as a colorless, crystalline powder. Yield: 34.27 g (82%). ^1^H NMR (C_6_D_6_, 400 MHz): δ 0.90 (t, OCH_2_C*H*_3_), 3.68 (q, OC*H*_2_CH_3_). ^13^C{^1^H} NMR (C_6_D_6_, 100.6 MHz): δ 13.47 (OCH_2_*C*H_3_), 66.84 (O*C*H_2_CH_3_). ^9^Be NMR (OEt_2_, 84.29 MHz): δ 5.06.

**Second step: Be(CH_3_)_2_ synthesis:** 3 M solution of CH_3_MgBr in Et_2_O (204 mL) was added dropwise to a solution of BeCl_2_(Et_2_O)_2_ (70 g, 0.306 mol) in Et_2_O (430 mL). After stirring the resulting mixture overnight, it was placed under reduced pressure to remove residual ether. The remaining crude solid was washed with benzene (3 × 125 mL) and placed under reduced pressure to remove residual benzene. Sublimation at 55–70 °C (0.1–0.2 Torr) was carried out over a period of 16–24 h to afford the desired product as a white powder. Yield: 10.32 g (86%). ^1^H NMR (C_5_D_5_N, 400 MHz): δ -0.28 (s, C*H*_3_). ^13^C{^1^H} NMR (C_5_D_5_N, 100.6 MHz): δ -2.39 (*C*H_3_). ^9^Be NMR (84.29 MHz): δ 22.69.


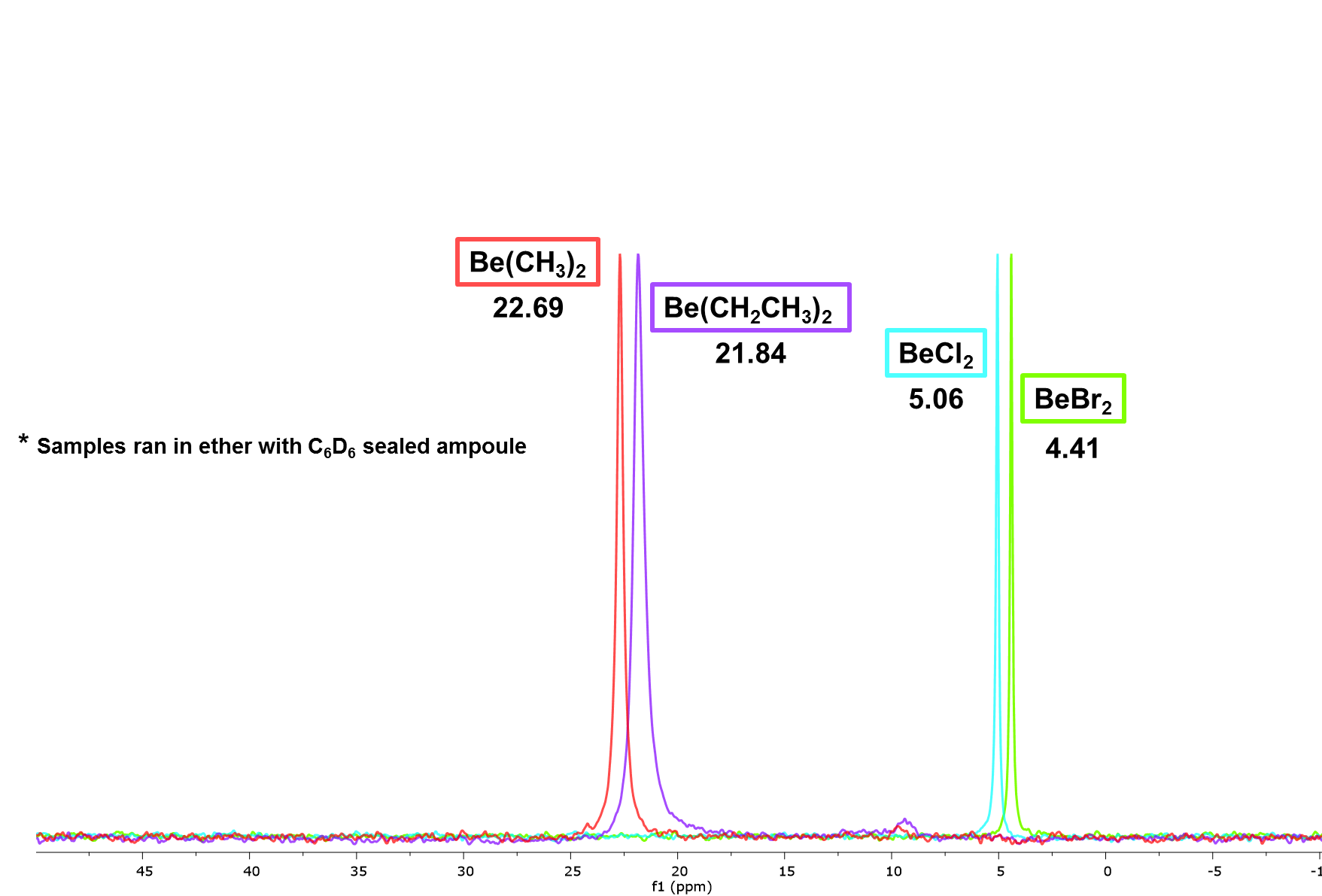


Figure S2. ^13^C{^1^H} NMR spectra of various Be precursors.

**2. High resolution TEM images**


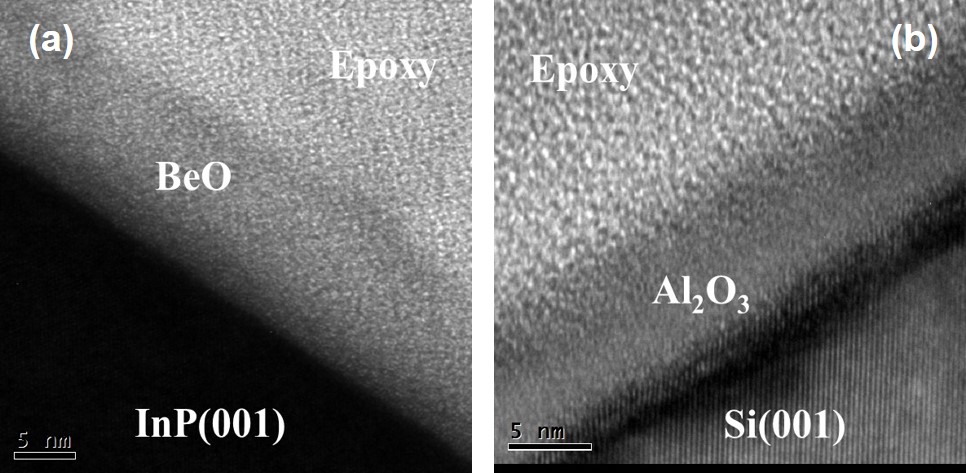


Figure S3. Cross sectional TEM image of (a) ALD BeO on InP and (b) ALD Al_2_O_3_ on Si.

ALD BeO grown on InP is amorphous unlike the ALD BeO grown on Si. ALD Al_2_O_3_ formed on Si is amorphous. Therefore, the high crystallinity of ALD BeO is strongly related to its lattice matching with the Si substrate.


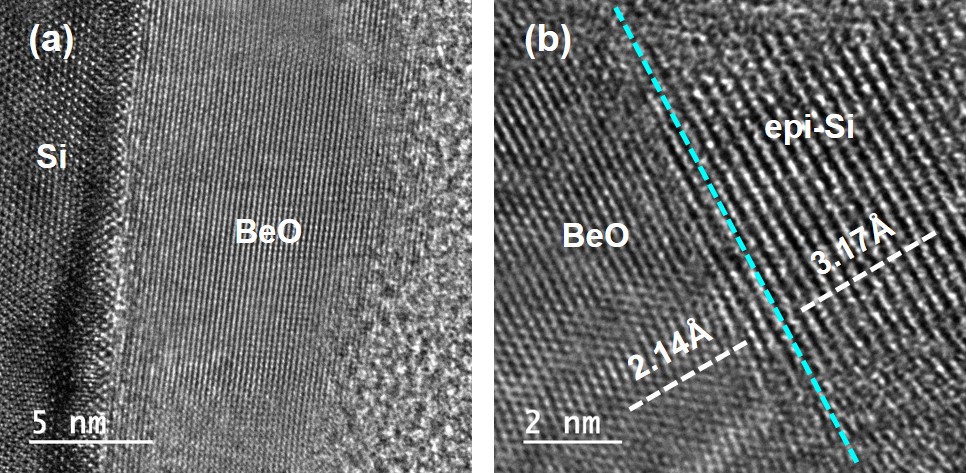


Figure S4. Cross sectional TEM image of (a) ALD BeO/Si (100) interface and (b) epi Si/BeO interface.

These images show clear interfaces of ALD BeO/Si (100) substrate and epi Si/ALD BeO.


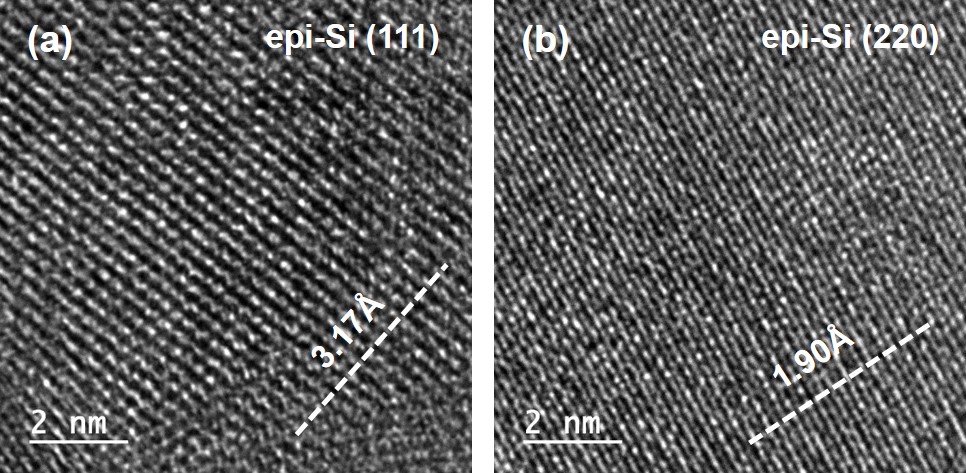


Figure S5. Cross sectional TEM image of epi (a) Si (111) and (b) Si (220)

The d-spacing values of epi Si (111) and (220) were mentioned in the figures.
